# True thymic hyperplasia causing pure red cell aplasia: a case report

**DOI:** 10.1093/icvts/ivab301

**Published:** 2021-11-13

**Authors:** Adam Mohammad, Alan G Dawson, Amrita Bajaj, Sridhar Rathinam

**Affiliations:** 1 Department of Thoracic Surgery, University Hospitals of Leicester NHS Trust, Leicester, UK; 2 Department of Radiology, University Hospitals of Leicester NHS Trust, Leicester, UK

**Keywords:** Pure red cell aplasia, True thymic hyperplasia, Thymectomy

## Abstract

Pure red cell aplasia caused by true thymic hyperplasia is extremely rare. We report the case of a 25-year-old female diagnosed with pure red cell aplasia. Following a thymectomy confirming true thymic hyperplasia and corticosteroid therapy, complete response was achieved. Patients diagnosed with pure red cell aplasia should be investigated with a computerized tomographic scan to assess for thymic pathology and if present, this should be resected. Follow-up is essential to monitor for recurrence.

## INTRODUCTION

Pure red cell aplasia (PRCA) is typified by a severe normochromic, normocytic anaemia associated with a reticulocytopaenia and an absence of erythroblasts from the bone marrow with preserved granulopoiesis and megakaryopoiesis [1]. In the acquired form, this can be primary or secondary to leukaemia, lymphoma, thymoma or solid tumours [1]. Thymoma has the strongest association with secondary PRCA, present in 7–10% of patients [2]. Thymic hyperplasia exists in two morphological forms: lymphofollicular thymic hyperplasia and true thymic hyperplasia [3, 4]. Lymphofollicular thymic hyperplasia is defined as the presence of a hyperplastic lymphoid germinal centre in the thymic medulla that is associated with a lymphocytic and plasma cell infiltration. There have been previously reported cases of PRCA in association with lymphofollicular hyperplasia in the literature [5, 6]. True thymic hyperplasia is defined as an increase in the size and weight of the thymus gland due to an increase in the number of epithelial cells. In addition, there is preservation of the original microscopic features. True thymic hyperplasia is a rarer cause with one previously described case [7]. We report a case of PRCA secondary to true thymic hyperplasia. 

## CASE REPORT

A 25-year-old female smoker presented with chest pain on exertion in August 2019. Other than a cholecystectomy, she had no other past medical history. Clinical examination was unremarkable and there were no recent medication changes. She had a severe normochromic, normocytic anaemia with a haemoglobin of 45 g/l, a red blood cell count of 1.68 × 10^12^/l, a reticulocyte count of 6 × 10^9^/l and a haematocrit of 0.131 l/l. Her white cell and platelet counts were 2.9 × 10^9^/l and 207 × 10^9^/l, respectively. An autoimmune antibody screen was negative. Her blood film was unremarkable and virology was negative for hepatitis B and C, human immunodeficiency virus, Epstein Barr Virus, cytomegalovirus and parvovirus B19. She was treated with regular blood transfusions, whilst a bone marrow aspiration and trephine biopsy confirmed a marked reduction in erythropoiesis in a reactive looking bone marrow with a normal myeloid series and megakaryocytes. The cytogenetic analysis confirmed a female karyotype without abnormality and a diagnosis of PRCA was made.

**Figure 1:**
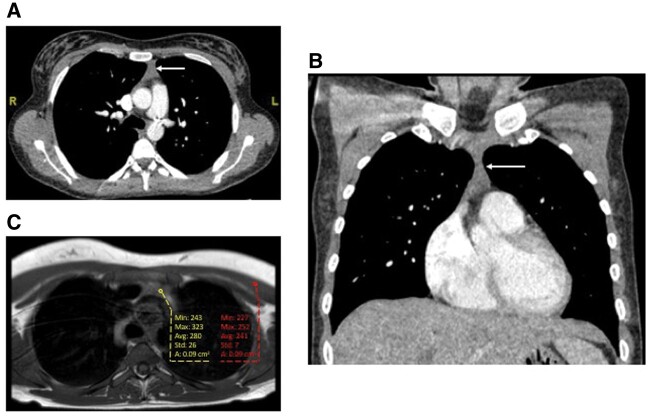
Computerized tomographic scan demonstrating anterior mediastinal soft tissue (arrow) in the (**A**) axial and (**B**) coronal planes. Axial T1W spin-echo magnetic resonance imaging scan (**C**) demonstrating anterior mediastinal soft tissue mildly hyperintense (yellow) compared to skeletal muscle control (red).

Computerized tomographic scanning of the thorax, abdomen and pelvis showed prominent triangular soft tissue in the anterior mediastinum (Fig. 1A and B), borderline splenomegaly and no systemic lymphadenopathy. A thoracic magnetic resonance imaging scan confirmed anterior mediastinal soft tissue with signal drop out on chemical shift imaging in keeping with thymic hyperplasia (Fig. 1C). She was referred to the thoracic surgical department and upon review provided informed consent for thymectomy.

**Figure 2:**
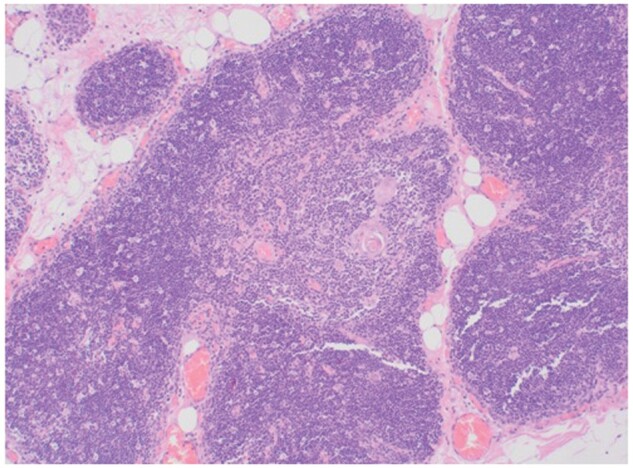
Haematoxylin and eosin stained thymus showing normal architecture. Magnification ×100.

In October 2019, thymectomy with total anterior mediastinal clearance via median sternotomy was performed. She made an uneventful recovery and was discharged on postoperative day 5. The resected specimen measured 80 × 74 × 4 mm with preserved lobular architecture and no evidence of reactive lymphoid hyperplasia (Fig. 2). True thymic hyperplasia was concluded. A 3-month reducing dose of Prednisolone was given.

At 21 months, she has not required any further immunosuppressive therapy or blood transfusions. Her haemoglobin, red blood cell count and haematocrit have normalized to 140 g/l, 4.78 × 10^12^/l and 0.413 l/l, respectively.

## DISCUSSION

True thymic hyperplasia causing PRCA is exceptionally rare; this is the second report in the literature and the first described in the UK. This case is unique in that after thymectomy and corticosteroid therapy, a complete response was achieved and maintained to 21 months.

The response of PRCA to thymectomy ranges from 25% to 30% [8]. This may be explained by the heterogeneous nature of thymic diseases. Owing to the low remission rate, there remains an appetite for adjuvant immunosuppressive therapy to facilitate disease control [2]. The previously reported case achieved complete response with surgical resection only [7]. Whilst in this case, complete remission may have been possible with thymectomy alone, corticosteroids were administered to optimize the probability of this. In the previous report, a thoracotomy was used to access the anterior mediastinum [7]; however, in this report, a median sternotomy was preferred as it offered maximal exposure allowing total anterior mediastinal tissue clearance. Whilst a less invasive video-assisted thoracoscopic surgery approach could have been employed, it was felt that this approach increased the risk of leaving thymic tissue behind which may be associated with the release of T-lymphocytes against erythroid precursors limiting the complete response achieved.

The improvement in erythropoiesis following thymectomy suggests an underlying pathophysiological mechanism and 2 have been proposed [1, 9]. The thymic abnormality may alter the subset of T-lymphocytes leading to production of autoimmune T-cell clones against erythroid precursors. Secondly, thymectomy may increase the risk of developing autoimmune disorders over time. In this case, the former would be a mechanistic explanation.

In conclusion, true thymic hyperplasia represents a rare diagnosis and accounts for a small proportion of thymic masses causing PRCA. Thymectomy and a short course of corticosteroid therapy have resulted in a complete response. This success must be tempered by caution as it may recur and long-term follow-up is essential.

**Conflict of interest:** none declared. 

## Reviewer information

Interactive CardioVascular and Thoracic Surgery thanks Toyofumi F. Chen-Yoshikawa and the other anonymous reviewers for their contribution to the peer review process of this article.
