# Corrigendum to ‘True thymic hyperplasia causing pure red cell aplasia: a case report’ [Interact CardioVasc Thorac Surg 2021; https://doi.org/10.1093/icvts/ivab301]

**DOI:** 10.1093/icvts/ivab374

**Published:** 2022-01-13

**Authors:** Adam Mohammad, Alan G Dawson, Amrita Bajaj, Sridhar Rathinam

**Affiliations:** 1 Department of Thoracic Surgery, University Hospitals of Leicester NHS Trust, Leicester, UK; 2 Department of Radiology, University Hospitals of Leicester NHS Trust, Leicester, UK

In the original version of this article, the allocation of the colours in the legend of Figure 1 was reversed. The correct legend should read as below and has now been corrected in the full article online.

**Figure 1:** Computerized tomographic scan demonstrating anterior mediastinal soft tissue (arrow) in the (**A**) axial and (**B**) coronal planes. Axial T1W spin-echo magnetic resonance imaging scan (**C**) demonstrating anterior mediastinal soft tissue mildly hyperintense (yellow) compared to skeletal muscle control (red).

